# Supplementary material for: Regulation of the hippocampal translatome by Apoer2-ICD release
Source: Mol Neurodegener. 2023 Sep 19;18:62. doi: 10.1186/s13024-023-00652-1 (PMC10510282; doi:10.1186/s13024-023-00652-1)
Supplement: Supplementary file 1 — Additional file 1: Figure S1. Regulation of Apoer2-ICD promoter-binding and effects of Apoer2-ICD mutations on Reln-promoter binding in vitro. Figure S2. Volcano plots of key TRAP-Seq comparisons. Figure S3. Transcription factors similarly regulated in Apoer2cKO and cleavage-deficient hippocampi. Figure S4. Overlapping overall transcript changes by genotype. Figure S5. Functional enrichments for all conditions by genotype. Figure S6. Majority of altered transcripts are specifically neuronal. [file 13024_2023_652_MOESM1_ESM.pdf]

Supplemental Figure 1

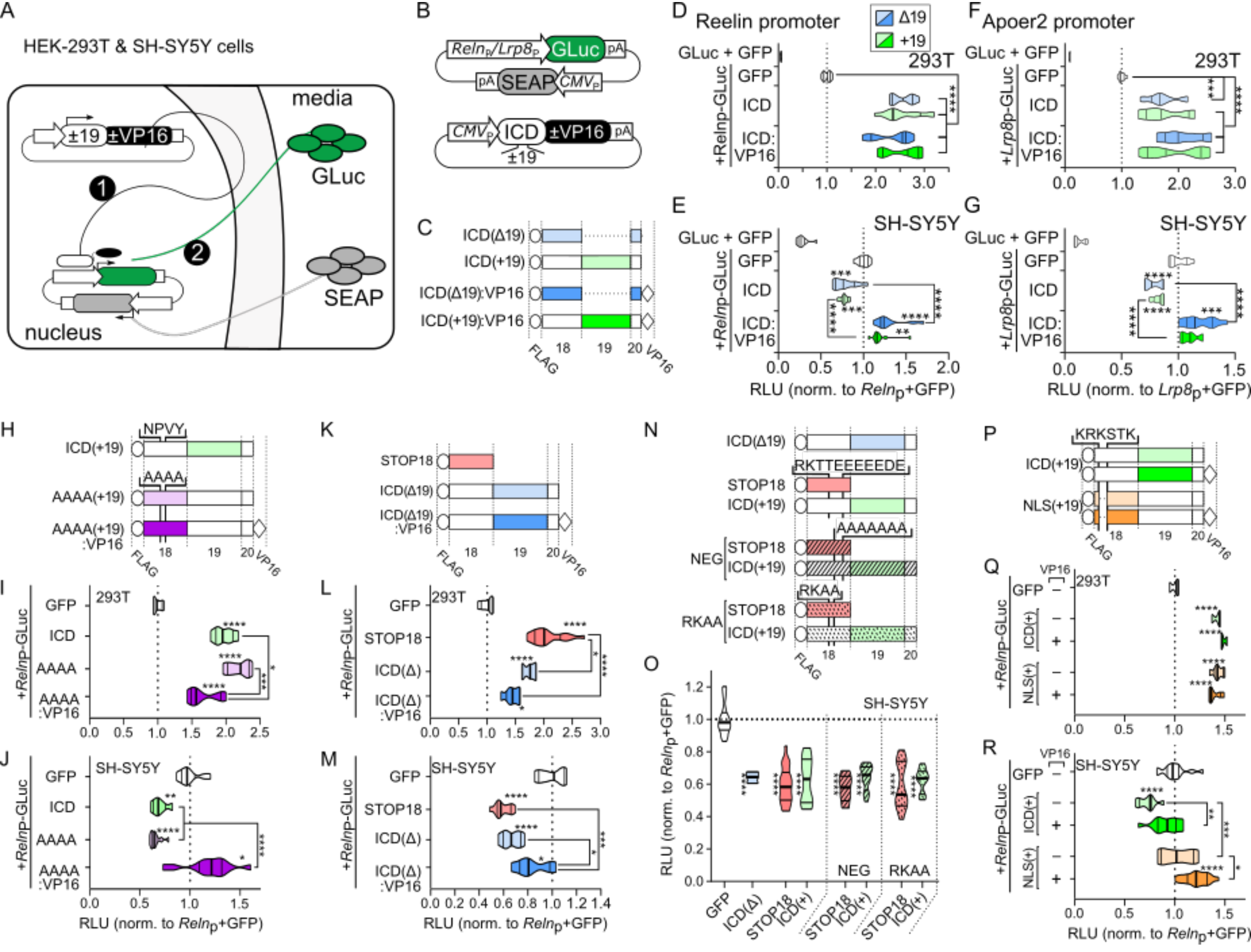

A
HEK-293T & SH-SY5Y cells
media
±19
±VP16
GLuc
SEAP
nucleus
B
Relnp/Lrp8p
GLuc
pA
SEAP
CMVp
ICD
±VP16
±19
C
ICD(Δ19)
ICD(+19)
ICD(Δ19):VP16
ICD(+19):VP16
FLAG
18
19
20
VP16
D
Reelin promoter
Δ19
+19
GLuc + GFP
293T
+Relnp-GLuc
GFP
ICD
ICD:
VP16
E
SH-SY5Y
RLU (norm. to Relnp+GFP)
F
Apoer2 promoter
+Lrp8p-GLuc
G
RLU (norm. to Lrp8p+GFP)
H
NPVY
AAAA
ICD(+19)
AAAA(+19)
AAAA(+19):VP16
I
J
AAAA
AAAA:VP16
K
STOP18
ICD(Δ19)
ICD(Δ19):VP16
L
M
ICD(Δ)
ICD(Δ):VP16
N
RKTTEEEEEDE
AAAAAAA
RKAA
NEG
O
RLU (norm. to Relnp+GFP)
GFP
ICD(Δ)
STOP18
ICD(+)
P
KRKSTK
NLS(+19)
Q
VP16
ICD(+)
NLS(+)
R

Figure S1: Regulation of Apoer2-ICD promoter-binding and effects of Apoer2-ICD mutations on *Reln*-promoter binding *in vitro*.

(**A-B**) Schematic representation of the luciferase assay in HEK-293T and the neuron-like SH-SY5Y cells (**A**) co-transfected with an Apoer2-ICD (±19±VP16) and a dual reporter construct containing the GLuc gene with the *Reln* or *Lrp8* promoter and CMV-driven (constitutive) control reporter (SEAP) (**B**). The transfected Apoer2-ICD (A1) enters the nucleus and either represses or drives GLuc expression (A2). (**C**) Schematic diagram of the Apoer2-ICD constructs. (**D-G**) Violin plots representing the distribution of GLuc expression driven by Apoer2-ICD binding to either the *Reln* (**D-E**) or *Lrp8* (**F-G**) promoters in HEK-293T (**D,F**) or SH-SY5Y cells (**E,G**).

(**H,K,N,P**) Diagrams of Apoer2-ICD constructs with N-terminal Flag tags (ellipse) ± VP-16 (diamond) depicting mutations tested in the panels immediately below. (**I-J, L-M, O, Q-R**) Violin plots representing the distribution of GLuc expression driven by Apoer2-ICD binding to the *Reln* promoter. (**H-J**) Effect of NPxY→ AAAA in HEK-293T (**I**) or SH-SY5Y (**J**). (**K-M**) Effect of STOP18 (no exon 19 or 20) in HEK-293T (**L**) or SH-SY5Y (**M**). (**N-O**) Mutations of negative charged amino acids (NEG) and potential tyrosine phosphorylation sites (RKAA) SH-SY5Y (**O**). (**P-R**) Mutation of the NLS in HEK-293T (**Q**) or SH-SY5Y (**R**). (Statistic details: One-way ANOVA [Tukey's post hoc multiple comparisons]. Panel D: n= 10 transfections, 2 independent experiments, F (5, 54) = 130.6, p<0.0001 [****p<0.0001], Panel E: n= 10 transfections, 3 independent experiments, F (5, 54) = 98.81, p< 0.0001 [**p=0.005, ***p=0.0009, ****p<0.0001], Panel F: n= 9 transfections, 2 independent experiments, F (5, 48) = 50.69, p< 0.0001 [***p<0.0002, ****p<0.0001], Panel G: n= 9 transfections, 2 independent experiments, F (5, 48) = 186.4, p<0.0001 [***p=0.0008, ****p<0.0001], Panel I: n= 6 transfections, 2 independent experiments, F (3, 20) = 59.16, p<0.0001 [*p=0.0202, ***p=0.0001, ****p<0.0001], Panel J: n= 6-12 transfections, 2 independent experiments, F (3, 36) = 31.84, p<0.0001 [*p=0.0189, **p=0.0021, ****p<0.0001], Panel L: n= 6-18 transfections, 2 independent experiments, F (3, 32) = 40.94, p<0.0001 [*p=0.0189, **p=0.0063, ****p<0.0001], Panel M: n= 6-12 transfections, 2 independent experiments; F (3, 26) = 28.41,

$p<0.0001$ [$^{*}p<0.03$, $^{***}p=0.0001$, $^{****}p<0.0001$], Panel O: $n=$ 3-24 transfections, 4 independent experiments, $F(7, 87) = 27.76$, $p<0.0001$ [$^{****}p<0.0001$], Panel Q: $n=$ 3-6 transfections, 1 independent experiments, $F(4, 16) = 44.76$, $p<0.0001$ [$^{****}p<0.0001$], Panel R: $n=$ 5-19 transfections, 3 independent experiments, $F(4, 56) = 28.62$, $p<0.0001$ [$^{*}p=0.0135$, $^{***}p=0.0026$, $^{***}p=0.0005$, $^{****}p<0.0001$])

Supplemental Figure 2

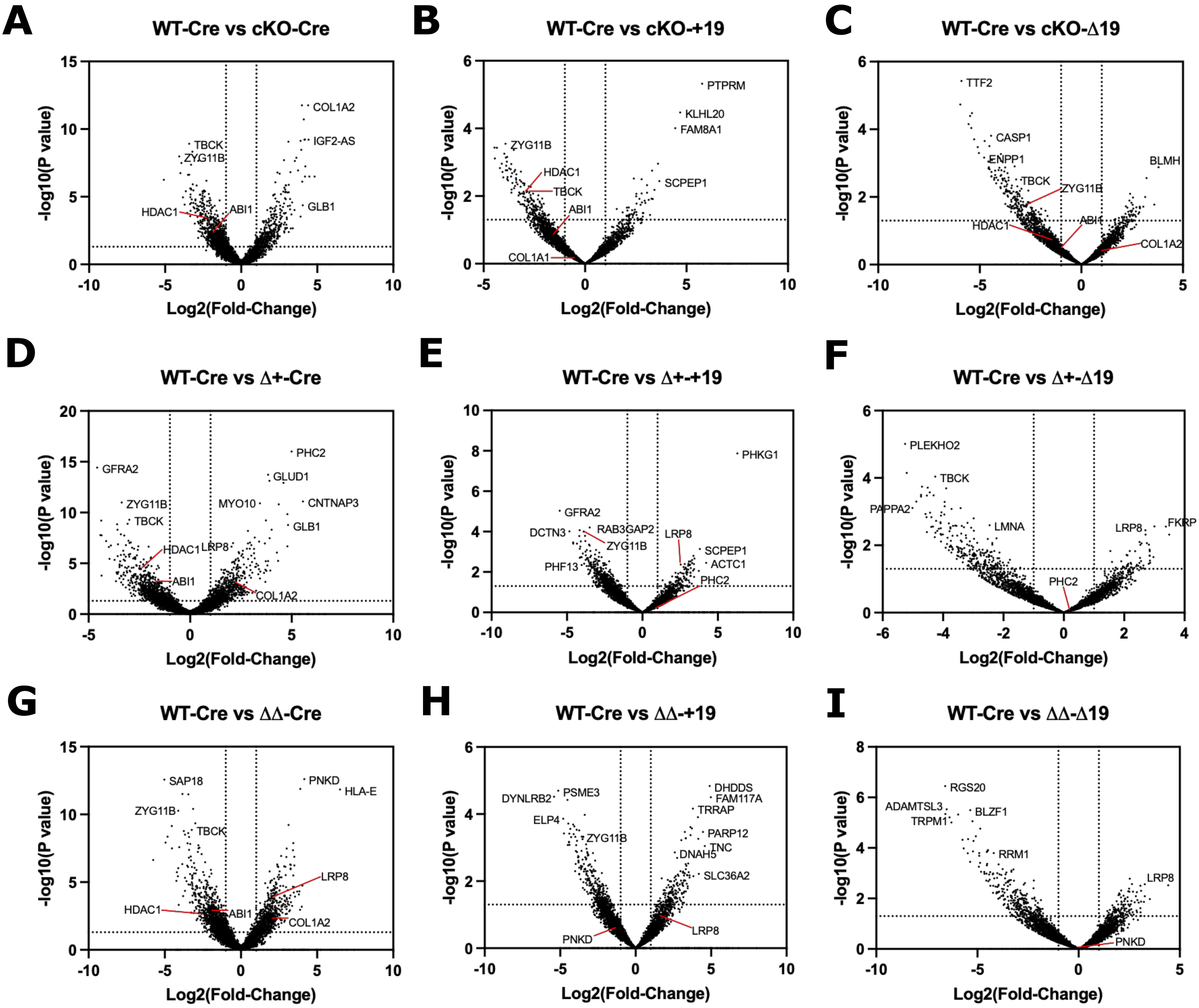
A
WT-Cre vs cKO-Cre
B
WT-Cre vs cKO-+19
C
WT-Cre vs cKO-Δ19
D
WT-Cre vs Δ+-Cre
E
WT-Cre vs Δ+-+19
F
WT-Cre vs Δ+-Δ19
G
WT-Cre vs ΔΔ-Cre
H
WT-Cre vs ΔΔ-+19
I
WT-Cre vs ΔΔ-Δ19
-log10(P value)
Log2(Fold-Change)
COL1A2
IGF2-AS
GLB1
TBCK
ZYG11B
HDAC1
ABI1
PTPRM
KLHL20
FAM8A1
SCPEP1
COL1A1
TTF2
CASP1
ENPP1
BLMH
PHC2
GFRA2
GLUD1
CNTNAP3
MYO10
LRP8
PHKG1
DCTN3
RAB3GAP2
PHF13
ACTC1
PLEKHO2
PAPPA2
LMNA
FKRP
SAP18
PNKD
HLA-E
DYNLRB2
PSME3
ELP4
DHDDS
FAM117A
TRRAP
PARP12
TNC
DNAH5
SLC36A2
RGS20
ADAMTSL3
TRPM1
BLZF1
RRM1

Figure S2: Volcano plots of key TRAP-Seq comparisons.

Volcano plots demonstrating significant transcripts altered between (**A**) Apoer2^{WT}-Cre vs Apoer2^{cKO}-Cre, (**B**) Apoer2^{WT}-Cre vs Apoer2^{cKO}-+19, (**C**) Apoer2^{WT}-Cre vs Apoer2^{cKO}-Δ19, (**D**) Apoer2^{WT}-Cre vs Apoer2$^{\Delta16}$-Cre, (**E**) Apoer2^{WT}-Cre vs Apoer2$^{\Delta16}$-+19, (**F**) Apoer2^{WT}-Cre vs Apoer2$^{\Delta16}$-Δ19, (**G**) Apoer2^{WT}-Cre vs Apoer2$^{\Delta16\Delta19}$-Cre, (**H**) Apoer2^{WT}-Cre vs Apoer2$^{\Delta16\Delta19}$-+19, (**I**) Apoer2^{WT}-Cre vs Apoer2$^{\Delta16\Delta19}$-Δ19. Line at $y = 1.3$ represents $p<0.05$ and lines at $x = -1$ and $x = 1$ represent fold-changes of 0.5 and 2, respectively.

Supplemental Figure 3

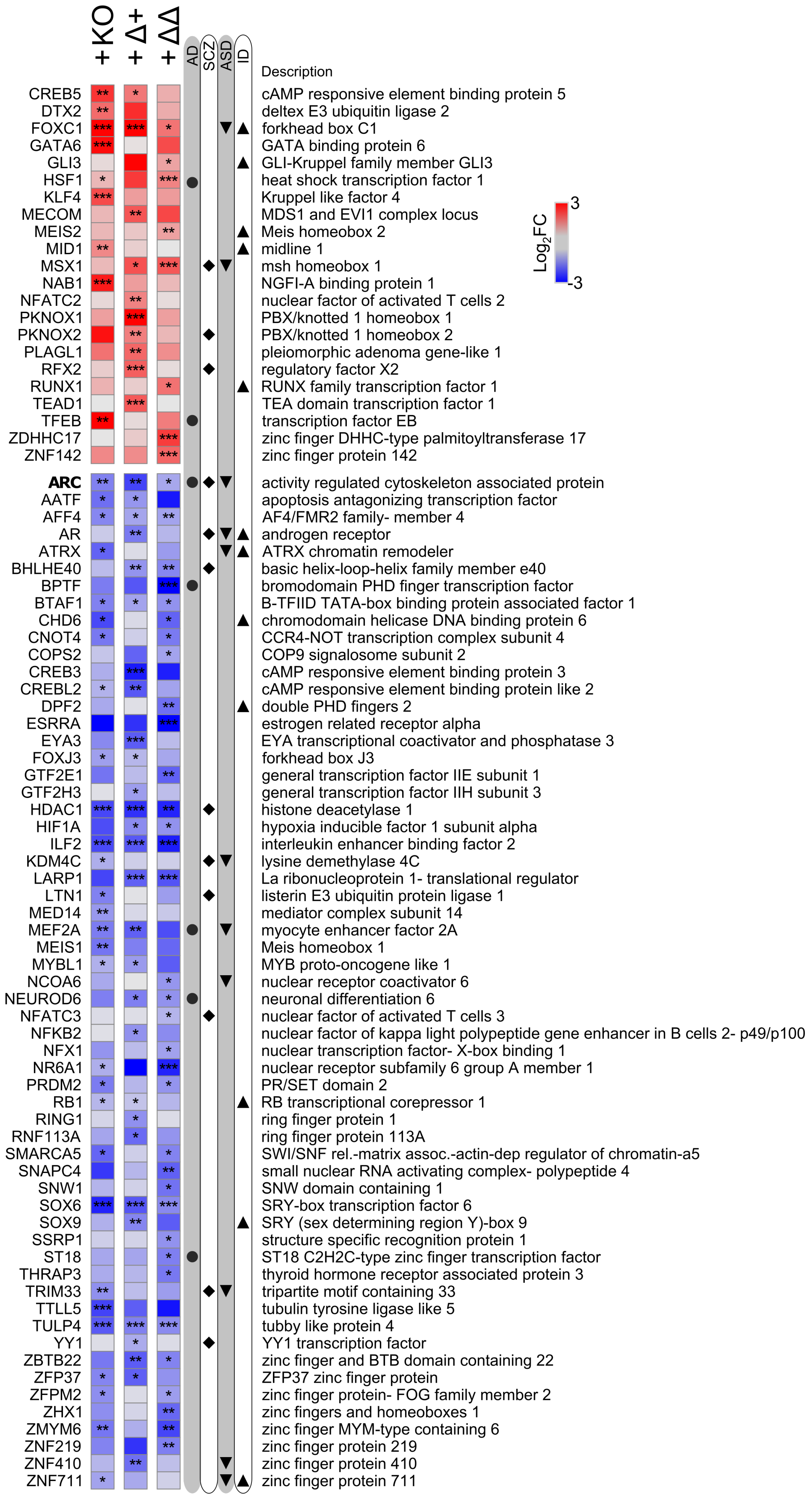

+KO
+Δ+
+ΔΔ
AD
SCZ
ASD
ID
Description
Log2FC
3
-3
CREB5 cAMP responsive element binding protein 5
DTX2 deltex E3 ubiquitin ligase 2
FOXC1 forkhead box C1
GATA6 GATA binding protein 6
GLI3 GLI-Kruppel family member GLI3
HSF1 heat shock transcription factor 1
KLF4 Kruppel like factor 4
MECOM MDS1 and EVI1 complex locus
MEIS2 Meis homeobox 2
MID1 midline 1
MSX1 msh homeobox 1
NAB1 NGFI-A binding protein 1
NFATC2 nuclear factor of activated T cells 2
PKNOX1 PBX/knotted 1 homeobox 1
PKNOX2 PBX/knotted 1 homeobox 2
PLAGL1 pleiomorphic adenoma gene-like 1
RFX2 regulatory factor X2
RUNX1 RUNX family transcription factor 1
TEAD1 TEA domain transcription factor 1
TFEB transcription factor EB
ZDHHC17 zinc finger DHHC-type palmitoyltransferase 17
ZNF142 zinc finger protein 142
ARC activity regulated cytoskeleton associated protein
AATF apoptosis antagonizing transcription factor
AFF4 AF4/FMR2 family- member 4
AR androgen receptor
ATRX ATRX chromatin remodeler
BHLHE40 basic helix-loop-helix family member e40
BPTF bromodomain PHD finger transcription factor
BTAF1 B-TFIID TATA-box binding protein associated factor 1
CHD6 chromodomain helicase DNA binding protein 6
CNOT4 CCR4-NOT transcription complex subunit 4
COPS2 COP9 signalosome subunit 2
CREB3 cAMP responsive element binding protein 3
CREBL2 cAMP responsive element binding protein like 2
DPF2 double PHD fingers 2
ESRRA estrogen related receptor alpha
EYA3 EYA transcriptional coactivator and phosphatase 3
FOXJ3 forkhead box J3
GTF2E1 general transcription factor IIE subunit 1
GTF2H3 general transcription factor IIH subunit 3
HDAC1 histone deacetylase 1
HIF1A hypoxia inducible factor 1 subunit alpha
ILF2 interleukin enhancer binding factor 2
KDM4C lysine demethylase 4C
LARP1 La ribonucleoprotein 1- translational regulator
LTN1 listerin E3 ubiquitin protein ligase 1
MED14 mediator complex subunit 14
MEF2A myocyte enhancer factor 2A
MEIS1 Meis homeobox 1
MYBL1 MYB proto-oncogene like 1
NCOA6 nuclear receptor coactivator 6
NEUROD6 neuronal differentiation 6
NFATC3 nuclear factor of activated T cells 3
NFKB2 nuclear factor of kappa light polypeptide gene enhancer in B cells 2- p49/p100
NFX1 nuclear transcription factor- X-box binding 1
NR6A1 nuclear receptor subfamily 6 group A member 1
PRDM2 PR/SET domain 2
RB1 RB transcriptional corepressor 1
RING1 ring finger protein 1
RNF113A ring finger protein 113A
SMARCA5 SWI/SNF rel.-matrix assoc.-actin-dep regulator of chromatin-a5
SNAPC4 small nuclear RNA activating complex- polypeptide 4
SNW1 SNW domain containing 1
SOX6 SRY-box transcription factor 6
SOX9 SRY (sex determining region Y)-box 9
SSRP1 structure specific recognition protein 1
ST18 ST18 C2H2C-type zinc finger transcription factor
THRAP3 thyroid hormone receptor associated protein 3
TRIM33 tripartite motif containing 33
TTLL5 tubulin tyrosine ligase like 5
TULP4 tubby like protein 4
YY1 YY1 transcription factor
ZBTB22 zinc finger and BTB domain containing 22
ZFP37 ZFP37 zinc finger protein
ZFPM2 zinc finger protein- FOG family member 2
ZHX1 zinc fingers and homeoboxes 1
ZMYM6 zinc finger MYM-type containing 6
ZNF219 zinc finger protein 219
ZNF410 zinc finger protein 410
ZNF711 zinc finger protein 711

Figure S3: Transcription factors similarly regulated in Apoer2^{cKO} and cleavage-deficient hippocampi.

Heatmap of the 81 transcription factors whose transcripts have altered similar differential ribosome-association in all three Apoer2 KI/cKO compared to Apoer2^{WT}.

Supplemental Figure 4

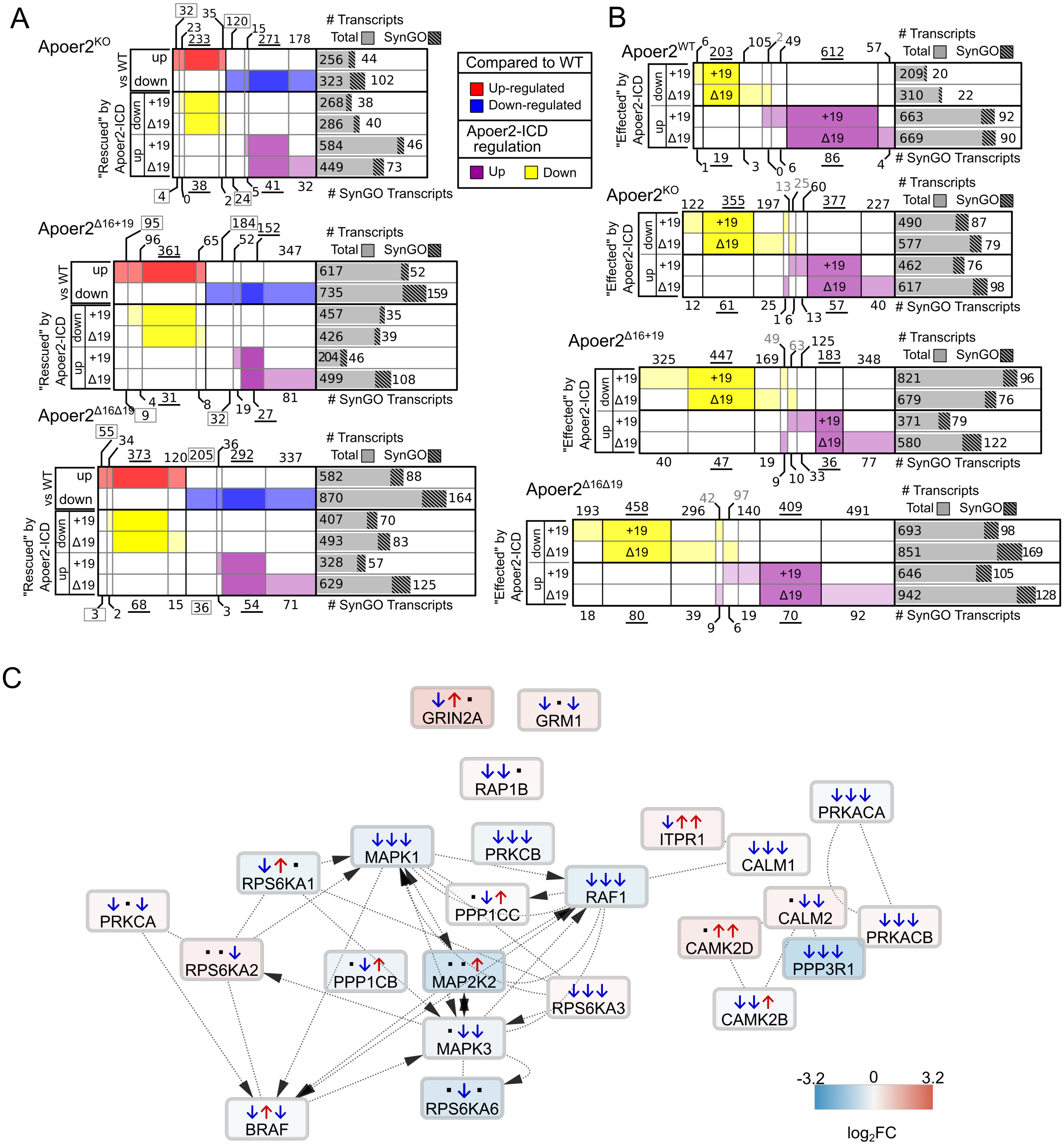
A
B
C
Apoer2KO
Apoer2Δ16+19
Apoer2Δ16Δ19
Apoer2WT
Transcripts
Total
SynGO
SynGO Transcripts
vs WT
up
down
"Rescued" by Apoer2-ICD
"Effected" by Apoer2-ICD
+19
Δ19
Compared to WT
Up-regulated
Down-regulated
Apoer2-ICD regulation
Up
Down
GRIN2A
GRM1
RAP1B
PRKACA
ITPR1
CALM1
MAPK1
PRKCB
RAF1
RPS6KA1
PPP1CC
CALM2
PRKACB
PRKCA
CAMK2D
PPP3R1
RPS6KA2
PPP1CB
MAP2K2
RPS6KA3
CAMK2B
MAPK3
RPS6KA6
BRAF
-3.2
0
3.2
log2FC

Figure S4: Overlapping overall transcript changes by genotype.

(**A-B**) Supervenns depicting the overlap of ribosome-associated transcripts altered in Apoer2 transgenic mice that are either rescued or not rescued with the Apoer2-ICD*[±19]* (**A**) or altered by overexpression of either Apoer2-ICD in Apoer2^{WT} or effects of the ICD independent of the lack of ICD-release (**B**). The numbers above indicate the total number of transcripts in common between the colored bars in the supervenn diagram, and below for SynGO transcripts. Boxes highlight the number of non-rescued transcripts. Underlined numbers are the number of transcripts regulated in the same direction by both Apoer2-ICD, while gray numbers indicate opposite directions. (**C**) Regulation of long-term potentiation. Diagram of the ribosome-bound transcripts altered in at least one Apoer2KI/KO compared to WT. Arrows above the transcript name indicate the direction of the change (up: red arrow, down: blue arrow) for each genotype (order: Apoer2^{cKO}, Apoer2$^{\Delta16+19}$, and Apoer2$^{\Delta16\Delta19}$). The fill color indicates the $\log_2$FC of these transcripts by the Apoer2-ICD in the WT.

Supplemental Figure 5

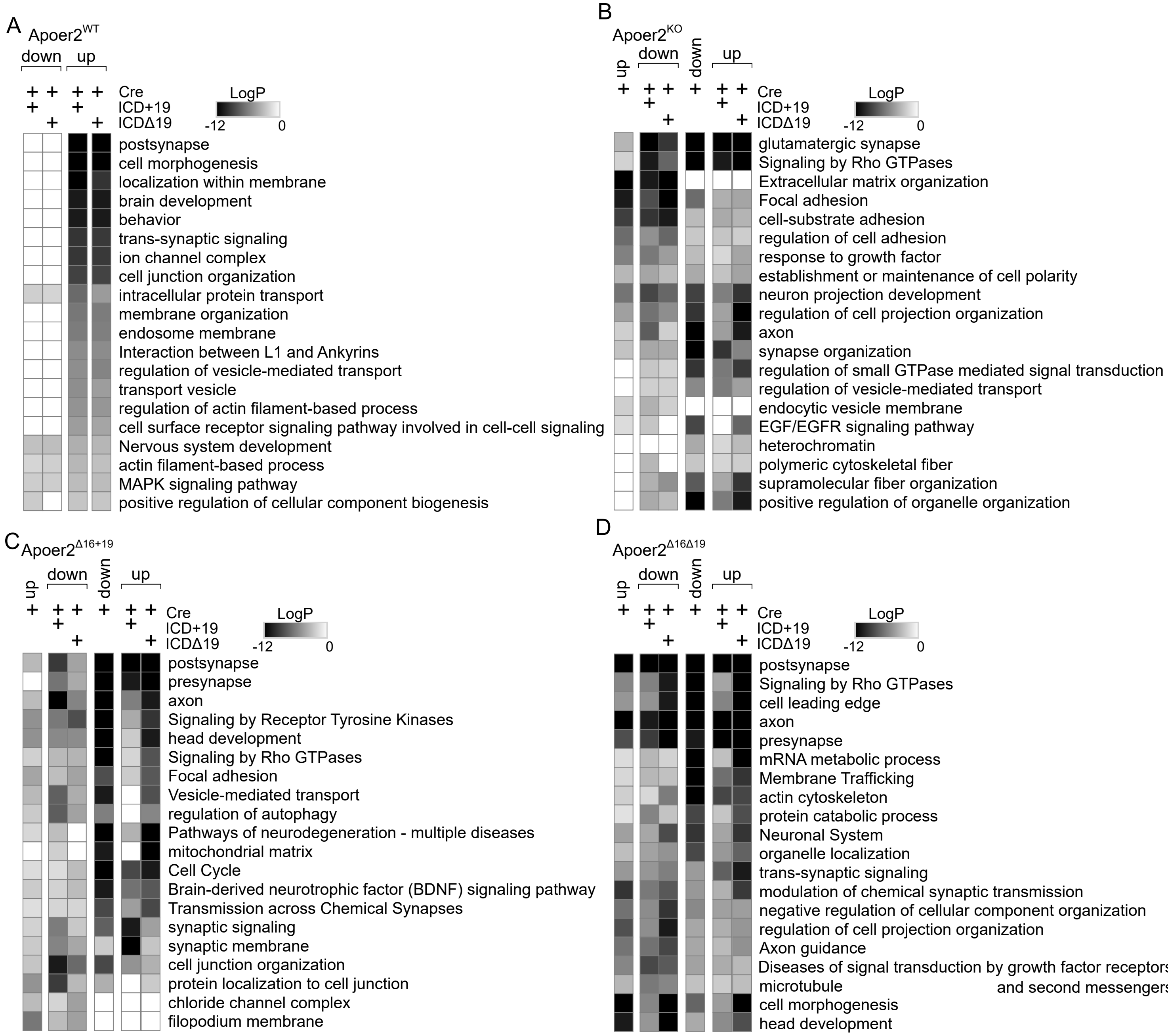
A
Apoer2^WT
down
up
Cre
ICD+19
ICDΔ19
LogP
-12
0
postsynapse
cell morphogenesis
localization within membrane
brain development
behavior
trans-synaptic signaling
ion channel complex
cell junction organization
intracellular protein transport
membrane organization
endosome membrane
Interaction between L1 and Ankyrins
regulation of vesicle-mediated transport
transport vesicle
regulation of actin filament-based process
cell surface receptor signaling pathway involved in cell-cell signaling
Nervous system development
actin filament-based process
MAPK signaling pathway
positive regulation of cellular component biogenesis
B
Apoer2^KO
up
down
down
up
Cre
ICD+19
ICDΔ19
LogP
-12
0
glutamatergic synapse
Signaling by Rho GTPases
Extracellular matrix organization
Focal adhesion
cell-substrate adhesion
regulation of cell adhesion
response to growth factor
establishment or maintenance of cell polarity
neuron projection development
regulation of cell projection organization
axon
synapse organization
regulation of small GTPase mediated signal transduction
regulation of vesicle-mediated transport
endocytic vesicle membrane
EGF/EGFR signaling pathway
heterochromatin
polymeric cytoskeletal fiber
supramolecular fiber organization
positive regulation of organelle organization
C
Apoer2^Δ16+19
up
down
down
up
Cre
ICD+19
ICDΔ19
LogP
-12
0
postsynapse
presynapse
axon
Signaling by Receptor Tyrosine Kinases
head development
Signaling by Rho GTPases
Focal adhesion
Vesicle-mediated transport
regulation of autophagy
Pathways of neurodegeneration - multiple diseases
mitochondrial matrix
Cell Cycle
Brain-derived neurotrophic factor (BDNF) signaling pathway
Transmission across Chemical Synapses
synaptic signaling
synaptic membrane
cell junction organization
protein localization to cell junction
chloride channel complex
filopodium membrane
D
Apoer2^Δ16Δ19
up
down
down
up
Cre
ICD+19
ICDΔ19
LogP
-12
0
postsynapse
Signaling by Rho GTPases
cell leading edge
axon
presynapse
mRNA metabolic process
Membrane Trafficking
actin cytoskeleton
protein catabolic process
Neuronal System
organelle localization
trans-synaptic signaling
modulation of chemical synaptic transmission
negative regulation of cellular component organization
regulation of cell projection organization
Axon guidance
Diseases of signal transduction by growth factor receptors and second messengers
microtubule
cell morphogenesis
head development

Figure S5: Functional enrichments for all conditions by genotype.

Functional enrichment for annotations found in the GO term biological processes and cellular components along with the pathway databases KEGG, REACTOME, and WIKIPATHWAYS in Apoer2^{WT} (**A**), Apoer2^{cKO} (**B**), Apoer2$^{\Delta 16+19}$ (**C**), Apoer2$^{\Delta 16 \Delta 19}$ (**D**).

Supplemental Figure 6

A

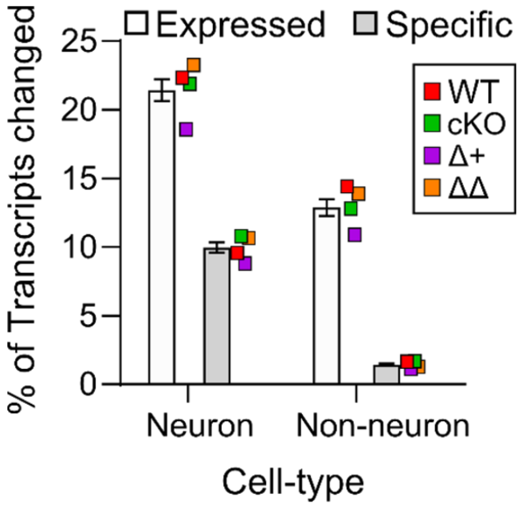

Expressed
Specific
WT
cKO
Δ+
ΔΔ
% of Transcripts changed
25
20
15
10
5
0
Neuron
Non-neuron
Cell-type

B

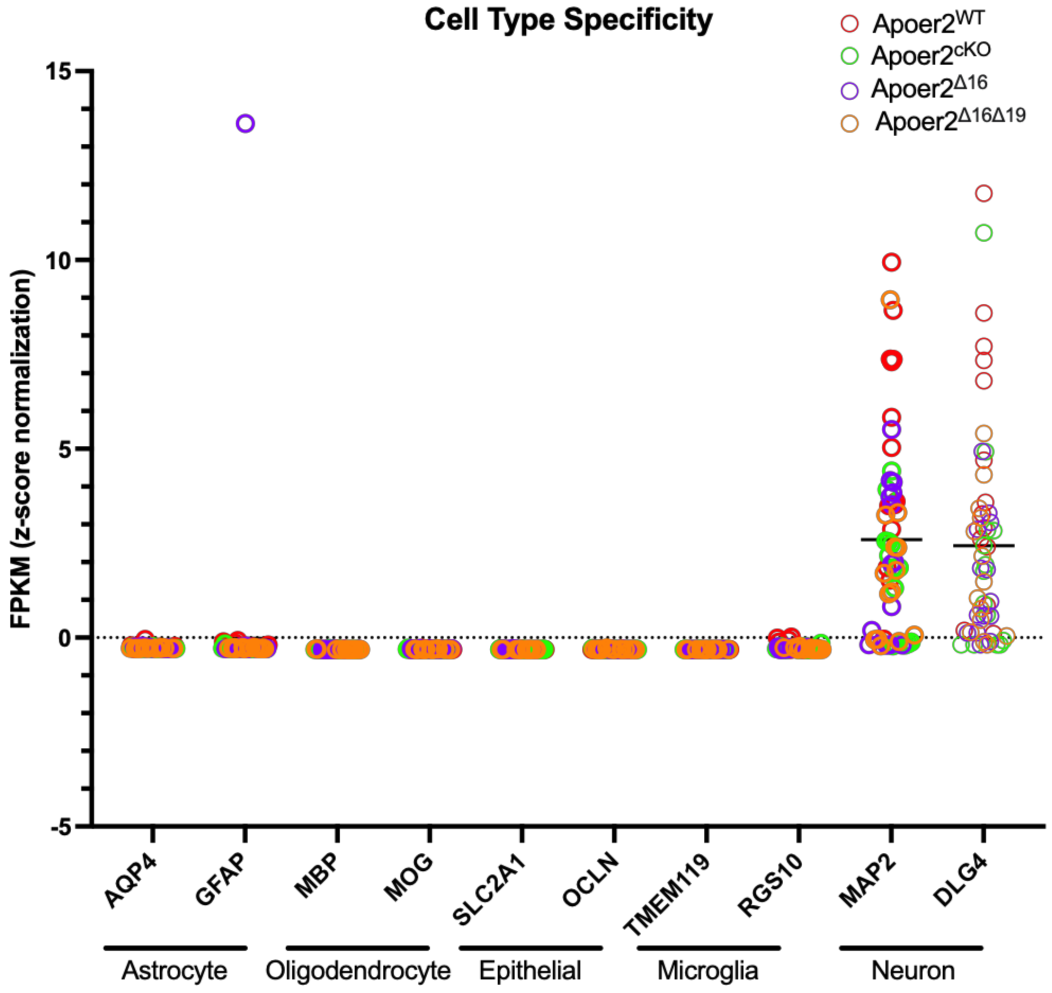

Cell Type Specificity
Apoer2WT
Apoer2cKO
Apoer2Δ16
Apoer2Δ16Δ19
FPKM (z-score normalization)
15
10
5
0
-5
AQP4
GFAP
MBP
MOG
SLC2A1
OCLN
TMEM119
RGS10
MAP2
DLG4
Astrocyte
Oligodendrocyte
Epithelial
Microglia
Neuron

Supplemental Figure S6: Majority of altered transcripts are specifically neuronal

(**A**) Percent of differentially regulated ribosome-associated transcripts in each genotype across conditions known to be expressed or expressed specifically in each hippocampal cell type from the Human protein atlas (Karlsson et al., 2021). (**B**) Standardized FPKM levels of astrocyte-, oligodendrocyte-, epithelial cell-, microglia-, and neuron-specific genes demonstrate TRAP-Seq primarily isolated neuronal transcripts.
